# High preoperative serum leptin level is an independent risk factor for deep vein thrombosis after total knee arthroplasty in osteoarthritis patients: A prospective and cross-sectional study

**DOI:** 10.1097/MD.0000000000010884

**Published:** 2018-05-25

**Authors:** Wanli Lu, Sheng Zhou, Long Xue, Bingyang Dai, Liang Qiao, Dongyang Chen, Zhihong Xu, Yao Yao, Huajian Teng, Qing Jiang

**Affiliations:** a Sports Medicine & Adult Reconstructive Surgery, Drum Tower Hospital, School of Medicine; b Laboratory for Bone and Joint Diseases, Model Animal Research Center, Nanjing University, Nanjing, Jiangsu, People's Republic of China.

**Keywords:** deep vein thrombosis, leptin, osteoarthritis, pulmonary embolism, total knee arthroplasty, venous thromboembolism

## Abstract

It suggests that a high leptin level may increase the risk of venous thromboembolism (VTE) in animal studies. However, clinical studies in this field are still largely unexplored. Our objective was to evaluate the relationship between the preoperative serum leptin levels and postoperative VTE incidence in osteoarthritis (OA) patients who underwent total knee arthroplasty (TKA) at our institute.

We conducted a prospective and cross-sectional study in these OA patients from March 2014 to March 2016. Preoperative leptin levels were analyzed by Luminex assays. VTE was assessed preoperatively and on postoperative day 5 and 7. The potential risk factors for VTE were also documented.

We enrolled 203 OA patients. No PE was detected and DVT was diagnosed in 34 patients postoperatively. There were significant differences between the median leptin levels in DVT group and non-DVT group [25.13 ng/mL (interquartile range, 14.51–44.31) vs 18.71 ng/mL (8.26–28.99), *P* = .007]. The relative risk of DVT significantly increased with natural logarithm (ln) leptin (per SD increase) (OR 2.37, 95% confidence interval (95% CI), 1.29–4.33, *P* = .005). Multivariate analyses adjusted for potential confounders showed ln leptin (per SD increase) was significantly associated with the relative risk of DVT (OR 2.17, 95% CI, 1.01–4.64, *P* = .046). When patients were subdivided into tertiles according to their leptin values, the OR for DVT increased with increasing tertiles of serum leptin (OR 1.03, 95% CI, 1.01–1.06, *P* for trend = .023).

In the present study, our results indicate that a high preoperative leptin level may be an independent risk factor for postoperative DVT.

## 1 Introduction

Venous thromboembolism (VTE), which comprises deep vein thrombosis (DVT) and pulmonary embolism (PE), is a major and potentially fatal complication after total knee arthroplasty (TKA).^[1]^ TKA is a common form of orthopedic surgery associated with improvements in the patients’ quality of life.^[2]^ Traditionally, the rate of DVT is estimated to be 30% to 40% in patients undergoing TKA without thromboprophylaxis.^[3]^ Although various thromboprophylactic measures have been introduced to reduce the incidence of these events, the incidence of in-hospital symptomatic DVT and PE after TKA still remains at 1.09% and 0.27%, respectively.^[4]^ Thus, the formation of VTE after TKA is still a challenge to orthopedic surgery.

Leptin, the product of the obese (ob) gene, is a ubiquitous 16-kDa pleiotropic protein primarily secreted by adipose tissue, which regulates adipose tissue mass and body weight.^[5–7]^ Leptin has multiple roles in the regulation of carbohydrate and lipid metabolism, reproductive system, inflammatory and immune reactions, cardiovascular and neurodegenerative diseases.^[8,9]^ Accumulating evidence suggest that leptin can also affect vascular homeostasis.^[10]^ Studies show that leptin may exert some protective effects, such as inducing nitric oxide production,^[11]^ promoting vascular regeneration.^[12]^ However, leptin has also been found to play a potential prothrombotic role, such as enhancing platelet adhesion, activation, and aggregation,^[13]^ promoting the formation of arterial thrombi,^[14]^ and inducing oxidative stress in endothelial cells.^[15]^ Positive associations of fibrinogen and clot lysis time with leptin was found in the lean and obese groups.^[16]^ Using a mouse model of induced VTE, a leptin-neutralizing antibody significantly reduced the formation of VTE and improved survival.^[17]^

In human, the possible role of leptin in the formation of VTE remains elusive to date. To our knowledge, no clinical study has been conducted to reveal the relationship between serum leptin concentration and VTE. We hypothesize that leptin may be a risk factor in the formation of postoperative VTE. Therefore, we conducted a prospective and cross-sectional study to examine the associations between preoperative leptin levels with VTE after TKA in osteoarthritis (OA) patients.

## 2 Materials and methods

### 2.1 Patient population and study design

A total of 263 OA patients who underwent primary unilateral TKA at the Department of Sports medicine & Adult reconstructive surgery, Drum Tower Hospital, School of Medicine, Nanjing University, from March 2014 to March 2016, were consecutively enrolled to this prospective and cross-sectional study. Scores were given to each knee radiograph according to the Kellgren–Lawrence (K-L) grade at admission.^[18]^ All patients received the lower extremities duplex ultrasonography preoperatively and bilateral venography postoperatively for evaluation of DVT. PE was diagnosed by computer tomography (CT) pulmonary angiography. Serum leptin levels of all patients were measured preoperatively by Luminex assays. Exclusion criteria was used to minimize confounders in the analysis, and included: Patients with immobility before surgery (>4 days); patients had a history of VTE and any contraindication to venography (e.g., allergy); patients underwent multiple joints replacements; patients positive for DVT on preoperative duplex ultrasonography; patients with comorbidities requiring anticoagulation, including previous malignancies and previously identified coagulation disorders; patients use of oral contraceptive pills or hormone replacement therapy; patients with cardiovascular diseases, such as coronary heart disease, congestive heart failure, and myocardial infarction; patients with preexisting stroke or postoperative infections.

In total, 60 patients were excluded and the remaining 203 OA patients (males/females: 39/164, the median age: 67 years (range 40–91 years)) were enrolled. The Ethical Committee of Drum Tower Hospital, School of Medicine, Nanjing University, approved the design of the study and all participants gave written informed consent according to the Declaration of Helsinki. The Strengthening the Reporting of Observational Studies in Epidemiology (STROBE) guidelines for reporting of observational studies were used to assess quality.^[19]^

### 2.2 Clinical data

Age, sex, VTE related history, and history of existing disease (such as: diabetes mellitus (DM), hypertension, coronary heart disease, congestive heart failure, myocardial infarction, stroke, renal and liver disease, medication taking, and malignant diseases) were recorded. We measured clinical and biochemical data (weight, height, serum lipids, and d-dimer) and surgery-related data (duration of surgery, volume of intraoperative blood loss). And BMI was calculated as the weight in kilograms divided by the square of height in meters.

### 2.3 Surgical procedures and postoperative management

All patients received general anesthesia, which was chosen by the patient and the anesthesiologist. The procedures were performed by a single surgical team following the same surgical technique. All the patients, which were checked with normal creatinine clearance when admitted to hospital, received identical DVT prophylaxis of rivaroxaban (Xarelto, Bayer, Leverkusen, Germany) 10 mg per day with a first-time oral dose given at 10 hours postsurgery and continued for at least 14 days postoperatively. The early active mobilization of the hip, knee, and ankle was started within 3 to 5 hours postoperatively. Intermittent pneumatic compression (Daesung Maref, Gunpo, Republic of Korea) of the lower extremities was encouraged as soon as possible postoperatively. On average, patients were discharged on the eighth postoperative day.

### 2.4 Assessment of VTE

Duplex ultrasonography examines were independently performed and diagnosed by 2 experienced examiners to assess the deep venous system from the inguinal ligaments to the ankle on the day before surgery.^[20]^ When patients were suspected of DVT in ultrasonography or suspected of PE, venography, or CT pulmonary angiography would be performed to confirm. As we described before, all the patients received bilateral venography on postoperative day 5 and diagnosed by 2 experienced doctors. PE was diagnosed by CT pulmonary angiography on postoperative day 7. If VTE was detected, conventional thrombolytic treatment was to be started.^[21,22]^ The proportion of patients with VTE (PE, proximal, or distal DVT) was calculated. DVT was classified as proximal if it involved the iliac, superficial femoral, or popliteal veins, with or without calf vein thrombosis, and as distal if it was isolated to the calf veins (e.g., posterior tibial, anterior tibial, peroneal, or gastrocnemius muscular branch veins).

### 2.5 Laboratory analyses

Peripheral venous samples were collected on the morning before surgery between 7:00 am and 8:00 am after an overnight fasting. All serum samples were obtained after centrifugation, then analyzed directly or frozen and stored at −80°C until thawed for Luminex assays. The specimens were analyzed in the clinical laboratory of Drum Tower Hospital, School of Medicine, Nanjing University within 2 hours using a fully automatic biochemical autoanalyzer (Hitachi 7600-20, Hitachi, Tokyo, Japan), serum levels of triglycerides (TG), total cholesterol (TC), high-density lipoprotein cholesterol (HDL-C), and low-density lipoprotein cholesterol (LDL-C), C-reactive protein (CRP) were detected. The preoperative d-dimer test was measured with D-Dimer PLUS (Dade Behring Co., Deerfield, IL) on the instruments of CA-6000 (Sysmex Co., Kobe, Japan). Serum levels of leptin and tumor necrosis factor α (TNFα) were measured with customized Milliplex MAP Kits Human Bone Panel (EMD Millipore, Billerica, MA) following the manufacturer's protocol. Data were acquired on a FlexMAP-3D system by percent and analyzed by using XPonent 4.0 software and 5-parameter logistic regression analysis. Standards were run in parallel to allow quantification of individual factors. Reported values included those within the standard curve range and those calculated by the logistic regression analysis. Units for all analytes are in pg/mL of fluid. Average analytical coefficients of variation across control samples for these analytes ranged from 0.26% to 5.33%. Technicians performing Luminex assays were blinded to the identity of patients.

### 2.6 Statistics

Categorical variables were compared using Fisher exact test. Continuous variables which have normal distribution were expressed as mean ± standard deviations (SD) and compared using Student *t* test or one-way ANOVA, as appropriate. When the data were found not to follow a normal distribution, they were presented as medians with corresponding 25th and 75th percentiles and compared using the unpaired Mann–Whitney *U* test or Kruskal–Wallis test when appropriate. Receiver operating curve analysis was used to determine the area under the curve for leptin with postoperative DVT. Correlation analyses were performed by Spearman coefficient. Univariate logistic regression was performed to calculate odds ratios (OR) with the corresponding 95% confidence intervals for postoperative DVT. All candidate predictor variables associated with DVT in univariate logistic regression analysis with a *P*-value <.10 were incorporated into a multivariate logistic regression model with clinical risk parameters. For parameters with established cut-off values, variables were dichotomized according to their specific thresholds. A positive test was considered for TC > 221.2 mg/dL, HDL-C < 36.3 mg/dL, LDL-C > 119.9 mg/dL, CRP > 8 mg/L, d-dimer > 0.5 mg/L. There were no established reference values for leptin and TNFα. Therefore, leptin and TNFα were used as a continuous variable and standardized after logarithmic transformation to correct its skewed distribution. In addition, ORs were calculated for tertiles of leptin for all patients. Tests for trend were performed by testing model coefficients for leptin levels in 3 tertiles group coded as a continuous variable with values equal to the median for each tertile in univariate logistic regression analysis. The power calculation was performed using Empower(R) (www.empowerstats.com, X&Y Solutions, Inc., Boston, MA). Our study had ≥99.9% to 1 power to detect ORs of 2.17 to 2.57 for DVT after TKA. Statistical analyses were performed by using the Software Statistical Package Sciences (SPSS) for Windows version 22.0 (SPSS, Inc., Chicago, IL). For all tests, *P* values were 2-tailed which less or equal to 0.05 were considered significant.

## 3 Results

In total, 203 OA patients were enrolled to this prospective and cross-sectional study. Postoperatively, no PE was detected in any patient and DVT was diagnosed in 34 patients. No proximal DVT was detected (Table 1). The preoperative clinical parameters, laboratory values of the study patients are shown in Table 2. In 203 patients underwent TKA, the median leptin levels in serum were 19.84 ng/mL (interquartile range (IQR), 9.58–30.82) and the median BMI, 25.8 kg/m^2^ (IQR, 23.4–28.9). As expected, leptin levels correlated, albeit weakly, with BMI values (positive correlation, *r*_s_ = 0.318, *P* < .001) (Table 3). Men had significantly lower leptin values than women (5.53 ng/mL (2.55–9.73) vs 22.27 ng/mL (13.59–35.07), *P* < .001), whereas BMI values were significantly different between the genders (men, 24.22 kg/m^2^ (22.6–27.08) vs women, 26.04 kg/m^2^ (23.54–29.13), *P* = .027).

**Table 1:**
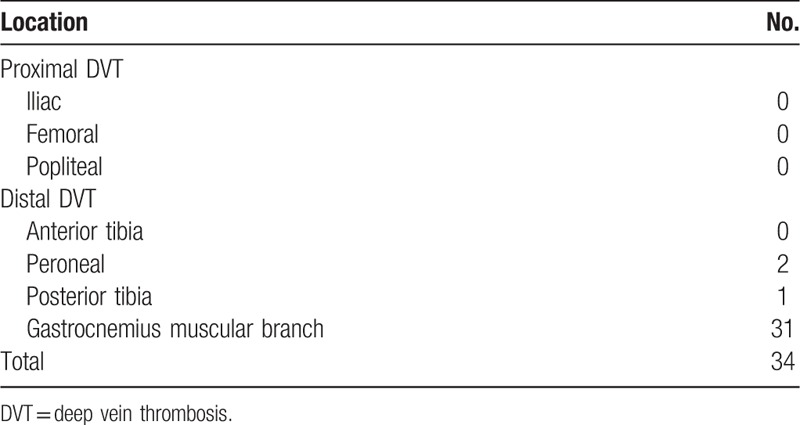
Distribution of DVT.

**Table 2:**
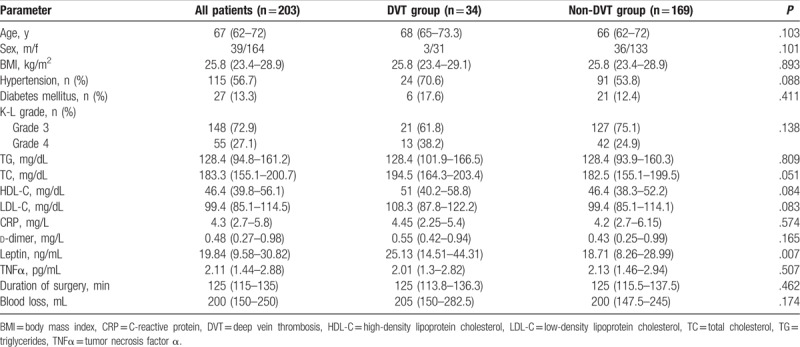
Preoperative characteristics of study patients and subgrouped by DVT development after TKA.

**Table 3:**
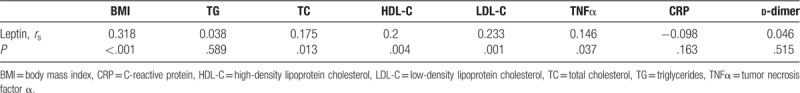
Spearman correlation coefficients of leptin levels with other parameters.

In subgroup analysis, the median leptin levels in DVT group and non-DVT group showed significant differences (*P* = .007) which were 25.13 ng/mL (14.51–44.31) and 18.71 ng/mL (8.26–28.99), respectively (Table 2). The values of TG, TC, HDL-C, LDL-C, CRP, TNFα, and d-dimer in DVT group showed no significant differences compared with those in non-DVT group. Also, no significant differences were observed in hypertension, diabetes mellitus, K-L scores, duration of surgery, and volume of intraoperative blood loss in the 2 groups. Receiver operating characteristic analysis further suggested that higher leptin levels preoperatively were associated with postoperative DVT with an area under the curve (AUC) of 0.65 (95% confidence interval (CI), 0.55–0.75). When leptin was analyzed as a continuous variable, there was a significant increase in the relative risk of DVT for every increase in the natural logarithm of leptin by one SD (OR 2.37, 95% CI (1.29–4.33), *P* = .005) (Table 4). Importantly, this effect was independent of BMI (OR for ln leptin adjusted by BMI; 2.68 (95% CI, 1.39–5.16), *P* = .003) and of age (OR for ln leptin adjusted by age; 2.35 (95% CI, 1.29–4.27), *P* = .005). Gender differences did not affect the odds of postoperative DVT with increasing leptin levels (OR for ln leptin for men, 2.64, 95% CI (0.54–13.05), *P* = .233; and for women, 0.93, 95% CI (0.5–1.74), *P* = .82). And, the number of patient's TC > 221.2 mg/dL, HDL-C < 36.3 mg/dL, CRP > 8 mg/L, d-dimer > 0.5 mg/L in the 2 groups did not affect the risk of postoperative DVT (Table 4). In a multivariate model of logistic regression analysis, ln leptin (per SD increase) was significantly associated with the relative risk of postoperative DVT (OR 2.17, 95% CI (1.01–4.64), *P* = .046), when adjusted for age, sex, BMI, hypertension, diabetes mellitus, d-dimer > 0.5 mg/L, LDL-C > 119.9 mg/dL, duration of surgery and intraoperative blood loss.

**Table 4:**
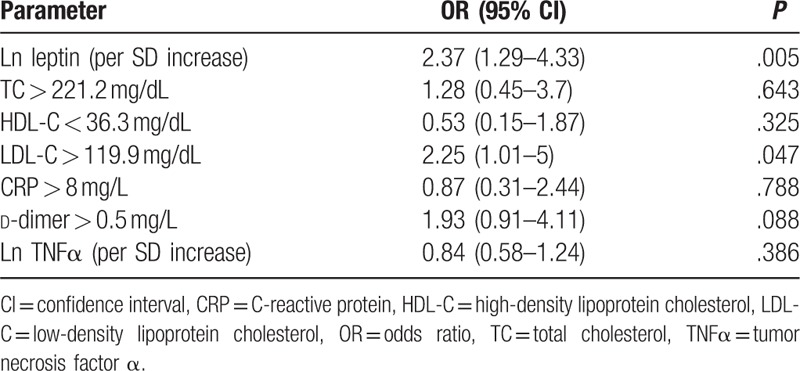
Univariate logistic regression examining the predictors of postoperative DVT.

Based on the tertiles of their leptin values (tertile 1, leptin < 12.19 ng/mL; tertile 2, leptin 12.19–25.91 ng/mL; tertile 3, leptin > 25.91 ng/mL), we classified patients into T1, T2, and T3 groups. No significant differences were found in age, diabetes mellitus, duration of surgery, blood loss and the levels of TG, TC, d-dimer, CRP, and TNFα among the 3 patient groups (Table 5). And, there were more women in the T3 group, corresponding to the higher median leptin values in women compared with men. In univariate logistic regression analysis, patients within T3 group had a 3.24-fold (95% CI, 1.18–8.89) increased risk of postoperative DVT compared with those in the T1 group (reference) (*P* = .022 for comparison of T3 vs T1). Of note, no differences were observed in the middle (T2 group, reference) and those in the highest leptin tertile (T3 group) with regard to the risk of postoperative DVT (*P* = .373). When leptin levels in T1–3 groups coded as a continuous variable with values equal to the median for each tertile the OR for DVT increased slightly with increasing tertile of serum leptin (OR 1.03, 95% CI (1.01–1.06), *P* for trend = .023).

**Table 5:**
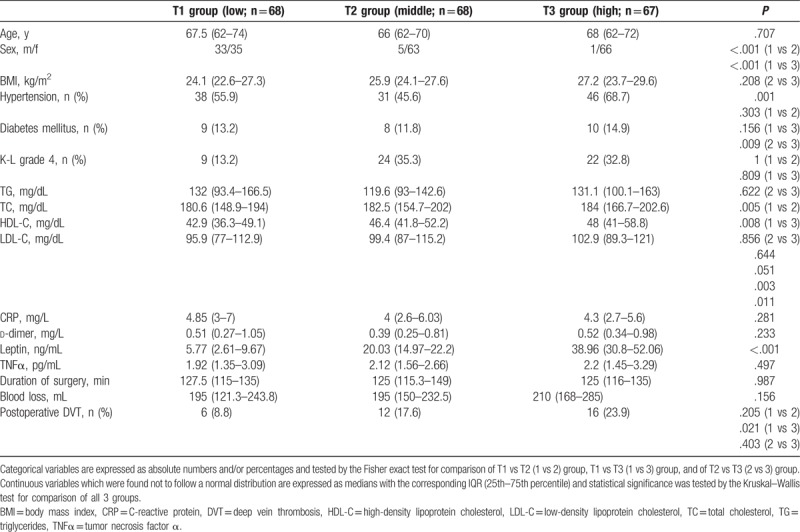
Demographic, clinical, and laboratory parameters, and outcome in the groups defined by the 3 leptin tertiles.

## 4 Discussion

In this prospective and cross-sectional study, which included 203 OA patients, we found that a high level of preoperative leptin was associated with postoperative DVT. This finding was consistent with the viewpoint of the prothrombotic effects of leptin in experimental studies.^[13,14,17,23,24]^ Few previous clinical studies have examined the association between leptin and VTE. In a case–control study involving 40 retinal vein occlusion (RVO) patients, the mean serum leptin levels were significantly higher in the patients with RVO than the control group.^[25]^ And a prospective study revealed that low plasma leptin concentration is a predictor for a complicated course and high mortality in patients with acute PE.^[26]^ Our study, to our knowledge, is the first to reveal that preoperative elevated serum leptin concentration is a risk factor for postoperative DVT which is independent of age, sex, BMI, hypertension, diabetes mellitus, d-dimer > 0.5 mg/L, LDL-C > 119.9 mg/dL, duration of surgery, and intraoperative blood loss. And with the increasing leptin levels in the T1–3 groups, the OR for DVT increased with increasing tertiles of serum leptin (*P* for trend = .023). So high serum leptin is an independent risk factor for postoperative DVT.

d-dimer, a predictive factor for VTE, is widely used in clinical practice as a diagnostic aid for suspected VTE.^[27,28]^ In our study, the preoperative values of d-dimer were mostly in normal range, showed no significant differences in DVT group and non-DVT group. And the results of duplex ultrasonography, bilateral venography and CT pulmonary angiography preoperatively were negative. These data indicated that all patients had no VTE preoperatively.^[20,29]^ Therefore, the selected patients are eligible for further research to reveal the relationship of leptin concentration and VTE.

In a retrospective study, which included 986 patients who underwent total hip arthroplasty (THA) or total or partial knee arthroplasty (TPKA), Fujita et al^[30]^ reported greater BMI and older patient age were independent risk factors for VTE after TPKA. In another retrospective study of 32,485 TKA patients, Wallace et al^[31]^ found that DVT/PE risk increased with obesity. However, in other studies, obesity was not found to be a risk factor for perioperative thromboembolic events in patients undergoing THA or TKA.^[32–35]^ In our study, we showed that leptin levels were associated with BMI, but the BMI itself was not a predictor of postoperative DVT, and the predictive value of leptin was independent of BMI values. Also, age was not a risk factor for DVT in our study. These findings may be due to the fact that we only enrolled the OA patients for TKA and the median BMI value and age were already elevated in our selected study population.

A case–control study which was carried out in men aged less than 55 years old with dyslipoproteinemia found that low levels of HDL-C were associated with VTE.^[36]^ We previously reported that a higher serum TG level was significantly associated with an increased DVT risk after THA in patients with nontraumatic osteonecrosis of the femoral head.^[37]^ However, in our study, the values of TG, TC, HDL-C, and LDL-C in DVT group showed no significant differences compared with those in non-DVT group. And the values of HDL-C and LDL-C in T1–3 groups were found significant differences which may be explained that they were positively correlated with leptin levels (Table 3). Because only 3 male DVT patients were included in this study, further gender differences in serum lipids and leptin were not conducted.

The elevated plasma inflammatory marks, such as CRP, TNFα levels have also been reported to be associated with increasing risk of DVT in case–control studies. However, this opinion is still in controversies.^[38–42]^ We found prospectively that the preoperative CRP, TNFα levels did not affect the risk of DVT after TKA in OA patients. In addition, we found that history of hypertension and diabetes mellitus, K-L grade scores, duration of surgery and intraoperative blood loss were not associated with the risk of DVT after TKA.

The present study used CT pulmonary angiography and bilateral venography to screen for PE and DVT following TKA, which ensured the accuracy and sensitivity of the diagnoses. However, it should be noted that our study has limitations. First, due to the more prevalence of OA in women,^[43,44]^ more women were included (males/females: 39/164) in the study. But gender differences did not affect the rate of DVT after TKA in DVT group and non-DVT group. Second, we investigated DVT and PE within a week after TKA, thereby it may underestimate the incidence of VTE.

In conclusion, our results show that high preoperative leptin level is a potential independent risk factor for postoperative DVT in this prospective and cross-sectional study. Leptin may play a role in the pathophysiology of DVT after TKA in OA patients. Cause–effect studies and deep understanding of the mechanisms are needed to target leptin to modify the venous status.^[45]^ Our study also suggests that inhibition of the prothrombotic effects of leptin may represent a novel therapeutic strategy for reducing DVT after TKA.

## Acknowledgment

We thank the patients and their families who donated their blood samples for this study.

## Author contributions

**Conceptualization:** Wanli Lu, Dongyang Chen, Zhihong Xu, Huajian Teng, qing jiang.

**Data curation:** Wanli Lu, Sheng Zhou, Bingyang Dai, Liang Qiao, Dongyang Chen, Yao Yao, qing jiang.

**Formal analysis:** Wanli Lu, qing jiang.

**Funding acquisition:** Huajian Teng, qing jiang.

**Investigation:** Wanli Lu, Sheng Zhou, Long Xue, Bingyang Dai, Liang Qiao, Yao Yao.

**Methodology:** Wanli Lu, Long Xue, Dongyang Chen, Zhihong Xu, Huajian Teng, qing jiang.

**Project administration:** qing jiang.

**Resources:** qing jiang.

**Software:** Wanli Lu.

**Supervision:** Dongyang Chen, Zhihong Xu, qing jiang.

**Validation:** Zhihong Xu, qing jiang.

**Writing – original draft:** Wanli Lu.

**Writing – review & editing:** Wanli Lu, Sheng Zhou, Huajian Teng, qing jiang.
